# Stereotactic Body Radiation Therapy for Prostate Cancer: What is the Appropriate Patient-Reported Outcome for Clinical Trial Design?

**DOI:** 10.3389/fonc.2015.00077

**Published:** 2015-03-31

**Authors:** Jennifer Ai-Lian Woo, Leonard N. Chen, Hongkun Wang, Robyn A. Cyr, Onita Bhattasali, Joy S. Kim, Rudy Moures, Thomas M. Yung, Siyuan Lei, Brian Timothy Collins, Simeng Suy, Anatoly Dritschilo, John H. Lynch, Sean P. Collins

**Affiliations:** ^1^Department of Radiation Medicine, Georgetown University Hospital, Washington, DC, USA; ^2^Department of Biostatistics, Bioinformatics, and Biomathematics, Georgetown University, Washington, DC, USA; ^3^Department of Urology, Georgetown University Hospital, Washington, DC, USA

**Keywords:** prostate cancer, SBRT, CyberKnife, EPIC, patient-reported outcome, toxicity

## Abstract

**Purpose:** Stereotactic body radiation therapy (SBRT) is increasingly utilized as primary treatment for clinically localized prostate cancer. Consensus regarding the appropriate patient-reported outcome (PRO) endpoints for clinical trials evaluating radiation modalities for early stage prostate cancer is lacking. To aid in clinical trial design, this study presents PROs over a 36-month period following SBRT for clinically localized prostate cancer.

**Methods:** Between February 2008 and September 2010, 174 hormone-naïve patients with clinically localized prostate cancer were treated with 35–36.25 Gy SBRT (CyberKnife, Accuray) delivered in 5 fractions. Patients completed the validated Expanded Prostate Cancer Index Composite (EPIC)-26 questionnaire at baseline and all follow-ups. The proportion of patients developing a clinically significant decline in each EPIC domain score was determined. The minimally important difference (MID) was defined as a change of one-half the standard deviation from the baseline. Per Radiation Therapy Oncology Group (RTOG) 0938, we also examined the patients who experienced a decline in EPIC urinary domain summary score of >2 points (unacceptable toxicity defined as ≥60% of all patients reporting this degree of decline) and EPIC bowel domain summary score of >5 points (unacceptable toxicity defined as >55% of all patients reporting this degree of decline) from baseline to 1 year.

**Results:** A total of 174 patients at a median age of 69 years received SBRT with a minimum follow-up of 36 months. The proportion of patients reporting a clinically significant decline (MID for urinary/bowel are 5.5/4.4) in EPIC urinary/bowel domain scores was 34%/30% at 6 months, 40%/32.2% at 12 months, and 32.8%/21.5% at 36 months. The patients reporting a decrease in the EPIC urinary domain summary score of >2 points was 43.2% (CI: 33.7%, 54.6%) at 6 months, 51.6% (CI: 43.4%, 59.7%) at 12 months, and 41.8% (CI: 33.3%, 50.6%) at 36 months. The patients reporting a decrease in the EPIC bowel domain summary score of >5 points was 29.6% (CI: 21.9%, 39.3%) at 6 months, 29% (CI: 22%, 36.8%) at 12 months, and 22.4% (CI: 15.7%, 30.4%) at 36 months.

**Conclusion:** Following prostate SBRT, clinically significant urinary symptoms are more common than bowel symptoms. Our prostate SBRT treatment protocol meets the RTOG 0938 criteria for moving forward to a Phase III trial comparing it to conventionally fractionated radiation therapy. Notably, between 12 and 36 months, the proportion of patients reporting a significant decrease in both EPIC urinary and bowel domain scores declined, suggesting a late improvement in these symptom domains. Further investigation is needed to elucidate (1) which EPIC domains bear the greatest influence on post-treatment quality of life and (2) at what time point PRO endpoint(s) should be assessed.

## Introduction

Stereotactic body radiation therapy (SBRT) is a new standard treatment option for clinically localized prostate cancer (1, 2). SBRT delivers high doses of radiation with precision to the prostate and adjacent tissues while minimizing radiation exposure to bladder and rectum (3, 4). Biochemical disease free survival has been shown to be high with SBRT (2, 5), demonstrating toxicity comparable to conventionally fractionated radiation therapy despite greater doses per fraction and higher biologically effective doses (5–8). Presently, evidence supporting superior outcomes associated with any particular radiation treatment approach for localized prostate cancer remains limited (9). As a result, the choice of intervention is guided by the treatment’s toxicity profile and the patient’s subsequent quality of life (QOL) (10).

Due to the close proximity of the bladder and rectum to the prostate, urinary and bowel toxicities are unavoidable following prostate cancer radiotherapy. These toxicities are commonly employed as the co-primary endpoints for Phase II trials evaluating the suitability of a new treatment option for clinically localized prostate cancer (11, 12). The clinical significance of these toxicities is determined by their severity, duration, and associated bother. Toxicity grade is clinician-assessed utilizing the items from the National Cancer Institute’s Common Terminology Criteria for Adverse Events (CTCAE). The incidence of late genitourinary (GU) and gastrointestinal (GI) toxicity (≥ grade 2) after external-beam radiation therapy ranges from 10 to 30%, generally occurring within the first 3 years. Recent data suggest that many low grade toxicities may resolve with time, and analysis of actuarial incidence may over-estimate their clinical significance (13).

Physicians’ assessment of treatment-associated toxicities is historically unreliable (14), and in fact may underestimate their severity (15, 16). Compared with physician-reported data, patient responses to validated questionnaires may better illustrate longitudinal trends in toxicity following radiotherapy (17). The Expanded Prostate Cancer Index Composite (EPIC)-26, a validated patient-reported outcome (PRO) instrument that evaluates health-related QOL, has been utilized to compare prostate cancer treatments with similar efficacy but differing toxicity profiles (10, 18, 19). Increasingly, PRO are integrated into clinical trial design (20), though interpretation of missing data (21, 22) and the selection of appropriate outcome measures complicate the meaningful use of PROs in the trial setting.

A key to utilizing PRO in clinical trials is determining thresholds for minimal important difference (MID) (23–25). An MID is the smallest difference in a questionnaire domain score, which patients perceive as a meaningful change (26). The MID for a given domain is important in determining the required number of patients for study recruitment and interpreting the questionnaire results. It varies depending on the specific domain questionnaire utilized and the demographics of the patient population being studied. The MID may be determined statistically or by comparison to results with a standard treatment (27). The most commonly used statistical approach is to utilize one-half SD of the baseline domain score (23), which is specific to the patient population being analyzed. Such a distribution approach has been criticized because it does not provide information on the clinical relevance of the observed change (26). In general, most approaches lead to MIDs that are 5–10% of the instrument range (23–25, 28).

What PRO endpoint should be utilized to determine if the toxicity profile of a new treatment is associated with a superior QOL? Radiation Therapy Oncology Group (RTOG) 0938 (http://www.rtog.org), a phase II trial comparing different SBRT hypofractionation regiments, compares urinary and bowel QOL 1 year following SBRT to that following conventionally fractionated external-beam radiation therapy (standard arm from RTOG 0415; 73.8 Gy in 41 fractions). In the opinion of the investigators, the percentage of patients with change in EPIC bowel domain score (baseline to 1-year) that was worse than five points and a change in EPIC urinary domain score (baseline to 1-year) that was worse than two points are felt to be clinically meaningful endpoints to assess for tolerability and safety. One year was chosen as a balance between a sufficient time to assess late toxicity with still adequate number of patients following up to minimize the impact of missing data.

To date, limited data are available on PROs following SBRT to aid in clinical trial design. The objective of this study is to report the urinary and bowel QOL outcomes following SBRT in patients with clinically localized prostate cancer. These PROs may in turn help inform selection of appropriate endpoints in the design of future clinical trials.

## Materials and Methods

### Patient selection

Eligible patients had a diagnosis of prostate cancer and were treated per our institutional protocol. Risk category was defined using the D’Amico classification (9). Patients who received androgen deprivation therapy (ADT) were excluded from this study due to its known adverse effects on PROs (29). Institutional IRB approval was obtained for retrospective review of data that were prospectively collected in our institutional database.

### SBRT treatment planning and delivery

Stereotactic body radiation therapy treatment planning and delivery were performed as previously described (4, 7). Gold fiducial markers were placed into the prostate using ultrasound guidance. Treatment plans were created using fused thin cut computed tomography (CT) images and high-resolution magnetic resonance (MR) images. The clinical target volume (CTV) included the prostate and proximal seminal vesicles. The planning target volume (PTV) included a 5 mm anterolateral expansion and a 3 mm posterior expansion around the CTV. A prescription dose of 35–36.25 Gy was delivered to the PTV in five fractions of 7–7.25 Gy over 1–2 weeks. The prescription isodose line was limited to ≥75%. The bladder, membranous urethra, and rectum were contoured and evaluated with dose–volume histogram analysis during treatment planning. Target position was confirmed multiple times during each treatment with a minimum of three properly placed fiducials (4).

### Follow-up and statistical analysis

Patients completed the EPIC-26 (30) before treatment and during routine follow-up visits 1 month after the completion of SBRT, every 3 months for the first year, and then every 6 months for the second and third years. To minimize the impact of missing data, patients who missed follow-ups were contacted and asked to fill out the questionnaires. The EPIC-26 is a validated tool that measures urinary and bowel QOL (30). To statistically compare changes between two time points, the levels of responses were assigned a score and the significance of the mean changes in the scores was assessed by paired *t*-test. Responses to the EPIC-26 questionnaire were grouped by physiological domains and assigned numerical scores. The multi-item scale scores were transformed linearly to a 0–100 scale as recommended in the scoring instructions for the EPIC-26. Lower numbers corresponded to worsening function and increased bother. Wilcoxon Signed-Rank Test analysis was used to assess differences in QOL scores compared to baseline. Paired *t*-test was used to assess significance of the change in scores. The MID to assess for clinically significant change in HRQOL from baseline was set as half an SD (23). Per RTOG 0938, we also examined the percentage of patients who experienced a decline in EPIC urinary domain summary score of >2 points (unacceptable toxicity defined as ≥60% of all patients reporting this degree of decline) and EPIC bowel domain summary score of >5 points (unacceptable toxicity defined as >55% of all patients reporting this degree of decline) from baseline to 1 year.

## Results

From February 2008 to September 2010, 174 hormone-naïve patients with clinically localized prostate adenocarcinoma were treated per our institutional SBRT monotherapy protocol. The patients were followed for a minimum of 36 months following SBRT (range: 37–69 months). The median patient age was 69 (48–90) years (Table 1). 55.7% of patients self-identified as white and 39.1% as black. Forty-two percent of patients were D’Amico low risk, 52.9% of patients were intermediate risk, and 5.1% of patients were high risk. The median prostate volume was 37.3 (11.6–138.7) cc. Moderate to severe lower urinary tract symptoms (baseline AUA ≥8, with a median baseline AUA of 7) were reported by 49.4% of patients prior to treatment (Table 2).

**Table 1:** **Patient characteristics at baseline**.

		Patients (*N* = 174)
**Age**	Age ≤60	13.80%
	60 < Age ≤ 70	46.60%
	Age >70	39.70%
**Race**	White	55.70%
	Black	39.10%
	Other	5.20%
**Median pre-treatment PSA (ng/mL)**		6.0 (1.8–32.5)
**Risk groups (D’Amico’s)**	Low risk	42.00%
	Intermediate risk	52.90%
	High risk	5.20%
**SBRT dose**	36.25 Gy	90.20%
	35 Gy	9.80%

**Table 2:** **Pre-treatment quality of life (QOL) scores**.

Baseline AUA score	% Patients (*n* = 174)
0–7 (mild)	50.6
8–19 (moderate)	44.8
>20 (severe)	4.6

**Baseline EPIC-26 summary score**	**Mean**	**SD**	**MID**

Urinary domain	89	11.06	5.5
Bowel domain	95	8.81	4.4

Ninety percent of patients were treated with 36.25 Gy in five 7.25 Gy fractions (Table 1). The median follow-up was 3.9 years. The median pre-treatment PSA of 6.0 ng/ml declined to a median 3 years post-treatment PSA of 0.3 ng/ml. There were six biochemical failures, occurring in one low-risk patient, four intermediate-risk patients, and one high-risk patient. The overall 3-year actuarial biochemical relapse free survival was 95.9%. No patients received ADT at any time during the first 3 years following SBRT.

**Figure 1:**
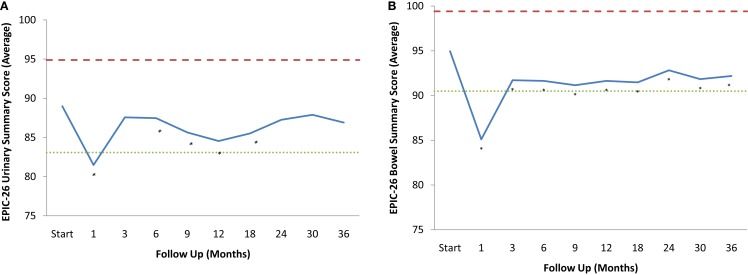
**EPIC Summary Scores**. **(A)** EPIC urinary summary domain scores at baseline and following SBRT for prostate cancer. **(B)** EPIC bowel summary domain scores at baseline and following SBRT for prostate cancer. Thresholds for clinically significant changes in scores (one-half SD above and below the baseline) are marked with dashed lines. EPIC scores range from 0 to 100 with higher values representing a more favorable health-related QOL.

**Figure 2:**
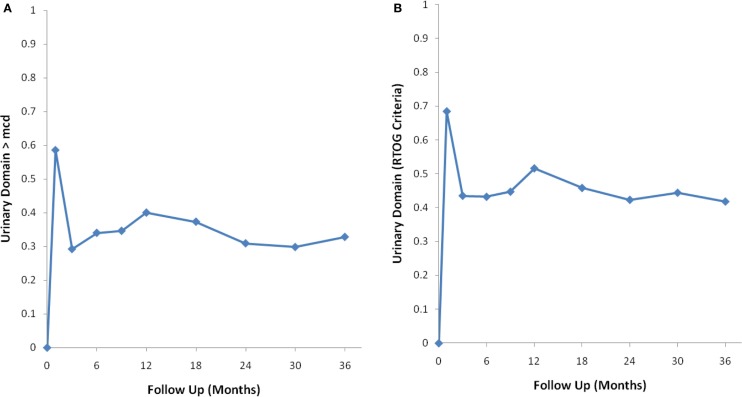
**Percentage of patients with a clinically significant decline in EPIC urinary summary domain scores following SBRT for prostate cancer**. **(A)** Clinically significant decline defined as a one-half SD below the baseline. **(B)** Clinically significant decline defined as a deviation in EPIC urinary domain summary score of >2 points below the baseline per RTOG 0938.

Baseline EPIC summary scores are shown in Table 2 and mean changes in EPIC summary scores from baseline to 3 years of follow-up are shown in Table 3. The MID value for the urinary domain was 5.5. The EPIC urinary summary score declined transiently at 1 month post-SBRT (mean change, −7.5) (Table 3; Figure 1A) and returned to near baseline by 3 months post-SBRT (mean change from baseline, −1.0) (Table 3; Figure 1A). This acute decline was both statistically (*p* < 0.0001) and clinically significant. A second late protracted decline occurred between 9 and 18 months (mean change from baseline at 12 months, −4.1) (Table 3; Figure 1A). The EPIC urinary summary score was close to baseline 3 years post-SBRT (mean change from baseline, −2.5) (Table 3; Figure 1A). The proportion of patients reporting a clinically significant decline in EPIC urinary domain scores was 34% at 6 months, 40% at 12 months, and 32.8% at 36 months (Table 4; Figure 2A). The patients reporting a decrease in the EPIC urinary domain summary score of >2 points was 43.2% (CI: 33.7%, 54.6%) at 6 months, 51.6% (CI: 43.4%, 59.7%) at 12 months, and 41.8% (CI: 33.3%, 50.6%) at 36 months (Table 5; Figure 2B).

**Table 3:** **Change in EPIC summary domain scores following SBRT for prostate cancer**.

Domain	1-month post-RT			3-month post-RT			12-month post-RT			24-month post-RT			36-month post-RT		
	Mean score			Mean score			Mean score			Mean score			Mean score		
	Change from baseline	SD	*p*	Change from baseline	SD	*p*	Change from baseline	SD	*p*	Change from baseline	SD	*p*	Change from baseline	SD	*p*
Urinary summary	−7.5	13.2	<0.0001	−1	10.8	0.200	−4.1	13.6	<0.0001	−1.7	14.9	0.097	−2.5	14.7	0.051
Bowel summary	−9.4	18.1	<0.0001	−3.0	12.0	0.0007	−2.9	11.4	<0.0001	−1.6	10.7	0.017	−2.4	13.1	0.004

**Table 4:** **Proportion of patients with clinically significant (> 0.5 SD) declines in EPIC-26 domain scores following SBRT for prostate cancer**.

	Start	1 month	3 months	6 months	9 months	12 months	18 months	24 months	30 months	36 months
**Urinary domain** (decrease >5.5 pts from baseline)	Median = 91.7 Mean = 89.3	58.5%	29.2%	34.0%	34.6%	40.0%	37.3%	30.9%	29.9%	32.8%
**Bowel domain** (decrease >4.4 pts from baseline)	Median = 100 Mean = 94.8	46.8%	24.4%	30.0%	29.4%	32.2%	24.3%	26.8%	29.7%	21.5%
*N*	174	171	168	162	159	155	142	149	144	134

**Table 5:** **Proportion of patients with decrements that met RTOG 0938 criteria for significant declines in EPIC-26 domain scores following SBRT for prostate cancer**.

	Start	1 month	3 months	6 months	9 months	12 months	18 months	24 months	30 months	36 months
**Urinary domain** (decrease >2 pts from baseline)	Median = 91.7Mean = 89.3	68.4%	43.5%	43.2% (33.7–54.6%)	44.7%	51.6% (43.4–59.7%)	45.8%	42.3% (34.3–50.7%)	44.4%	41.8% (33.3–50.6%)
**Bowel domain** (decrease >5 pts from baseline)	Median = 100 Mean = 94.8	46.8%	23.8%	29.6% (21.9–39.3%)	30.2%	29.0% (22.0–36.8%)	24.1%	24.8% (18.1–32.5%)	29.2%	22.4% (15.7–30.4%)
*N*	174	171	168	162	159	155	142	149	144	134

**Figure 3:**
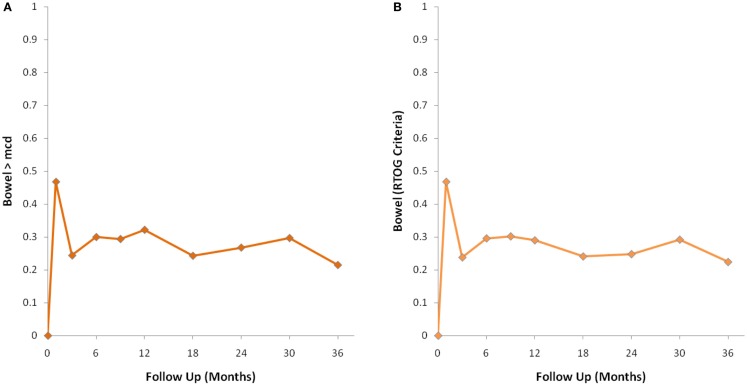
**Percentage of patients with a clinically significant decline in EPIC bowel summary domain scores following SBRT for prostate cancer**. **(A)** Clinically significant decline defined as a one-half SD below the baseline. **(B)** Clinically significant decline defined as a deviation in EPIC bowel domain summary score of >5 points below the baseline per RTOG 0938.

The MID value for the bowel domain was 4.4. The EPIC bowel summary score declined transiently at 1 month (mean change, −9.4) (Table 3; Figure 1B) and experienced a second, more protracted decline between 9 and 18 months (mean change from baseline at 12 months, −2.9). Bowel declines at 1 and 12 months were statistically significant (*p* < 0.0001); however, only the 1 month change met the threshold for clinically significant change. The EPIC bowel summary score were near baseline at 3 years post-SBRT (mean change from baseline, −2.4) (Table 3; Figure 1B). The proportion of patients reporting a clinically significant decline in EPIC bowel domain scores was 30% at 6 months, 32.2% at 12 months, and 21.5% at 36 months (Table 4; Figure 3A). The patients reporting a decrease in the EPIC bowel domain summary score of >5 points was 29.6% (CI: 21.9%, 39.3%) at 6 months, 29% (CI: 22%, 36.8%) at 12 months, and 22.4% (CI: 15.7%, 30.4%) at 36 months (Table 5; Figure 3B).

## Discussion

Post-treatment urinary and bowel QOL are important considerations in the management of clinically localized prostate cancer. Because SBRT is a newer management option for prostate cancer, longitudinal data reflecting urinary and bowel outcomes have yet to fully mature (1, 2). Expanded PROs in this area would facilitate improved clinical trial design and selection of appropriate early stage interventions.

Consensus is lacking regarding the appropriate PRO endpoints for clinical trials evaluating radiation modalities for early stage prostate cancer. The EPIC-26 is a commonly utilized prostate cancer-specific questionnaire (30); however, limited data are available to guide assessment of meaningful changes in EPIC-26 domain scores. Using a distribution approach, we found that the urinary domain had a higher MID value (5.5) than the bowel domain (4.4). Reassuringly, both MIDs were similar to those recently reported by others (28). The availability of these values should aid clinicians in utilization of the EPIC-26 for symptom management decisions.

In this study, we show that clinically significant urinary symptoms are more common than bowel symptoms over 36 months following prostate SBRT. Compared to RTOG 0415, the proportion of our patients with 1 year EPIC urinary domain declines >2 pts was higher (51.6 vs. 40%). However, the proportion of our patients with 1 year EPIC bowel domain declines >5 pts was lower (29 vs. 35%). Our patients were treated with 35 or 36.25 Gy in five fractions, which corresponds to a tumor equivalent dose in 2-Gy fractions (EQD2) of approximately 85–90 Gy assuming an α/β ratio of 1.5. Considering this high BED and our inhomogeneous treatment plans (31), it is not surprising that the percentage of our patients experiencing an EPIC urinary domain score decline was higher than patients treated with low dose conventionally fractionated intensity-modulated radiation therapy (IMRT) (73.8 Gy) at 1 year after treatment. Unexpectedly, the percentage of our patients experiencing an EPIC bowel domain score decline was lower than patients treated on the control arm of RTOG 0415. We believe that this favorable bowel QOL profile with SBRT may be secondary to increased accuracy with intrafraction fiducial tracking and narrowed target volumes, thus sparing normal rectum from inadvertent irradiation. Which treatment-related symptom bears the greatest influence on post-treatment QOL? Utility analyses have shown that bowel symptoms have a greater negative impact on QOL than urinary symptoms or impotence (32). However, this may not apply for all patients, and shared decision making may be most appropriate (33).

An important finding of this study is that our prostate SBRT treatment protocol meets the RTOG 0938 criteria, suggesting that urinary and bowel QOL is not significantly worse following our SBRT approach compared with conventionally fractionated IMRT. Based on patient preference for a shorter treatment course, SBRT utilization is likely to continue to increase as long as post-treatment QOL is comparable to conventionally fractionated IMRT.

At what time point should PRO endpoint(s) be assessed following prostate SBRT? Due to cost constraints, the timing of PRO assessments in Phase II trials are commonly limited to baseline and at one additional key time point that will determine whether to move the therapy forward to a Phase III trial. Acute toxicities usually resolve with time, but late toxicities commonly persist to cause a greater impact on long-term QOL. The length of follow-up (at least 36 months) in this cohort permitted us to capture a clinically meaningful difference in urinary and bowel symptoms, which may not be reflected by evaluating MID at the time point of 1 year per the existing RTOG protocol. Evaluation of PROs at a later time point beyond 1 year may yield more accurate assessment of long-term urinary and bowel QOL following radiation therapy. Recent evidence suggests that incorporation of web-based QOL survey technology in clinical trial design may further raise response rates, thus expanding opportunities to document even longer-term outcomes (17, 21).

## Conclusion

Following prostate SBRT, clinically significant urinary symptoms are more common than bowel symptoms. Our prostate SBRT treatment protocol meets the RTOG 0938 criteria for moving forward to a Phase III trial comparison to conventionally fractionated radiation therapy. Notably, between 12 and 36 months, the proportion of patients reporting a significant decrease in both EPIC urinary and bowel domain scores declined, suggesting a late improvement in these symptoms.

## Conflict of Interest Statement

Sean P. Collins and Brian Timothy Collins serve as clinical consultants to Accuray Inc. The Department of Radiation Medicine at Georgetown University Hospital receives a grant from Accuray to support a research coordinator. The other authors declare that they have no competing interests.

## Abbreviations

ADT, androgen deprivation therapy; CT, computed tomography; CTCAE, common terminology criteria for adverse events; CTV, clinical target volume; DVH, dose–volume histogram; EBRT, external-beam radiation therapy; EPIC, expanded prostate index composite; GI, gastrointestinal; GTV, gross target volume; GU, genitourinary; IMRT, intensity-modulated radiation therapy; MID, minimal important difference; PTV, planning target volume; QOL, quality of life; RTOG, radiation therapy oncology group; SBRT, stereotactic body radiation therapy; SD, standard deviation.
